# *Francisella tularensis* Bacteria Associated with Feline Tularemia in the United States

**DOI:** 10.3201/eid2012.131101

**Published:** 2014-12

**Authors:** Marilynn A. Larson, Paul D. Fey, Steven H. Hinrichs, Peter C. Iwen

**Affiliations:** University of Nebraska Medical Center, Omaha, Nebraska, USA

**Keywords:** feline-associated tularemia cases, Francisella tularensis, genotyping, domestic cats, Felis catus, bacteria, United States, vector-borne infections

## Abstract

Tularemia in the United States was examined by reviewing 106 *Francisella tularensis* isolates, mostly from Nebraska, collected during 1998–2012: 48% of Nebraska cases were cat-associated; 7/8 human cases were caused by subtype A.I. A vaccine is needed to reduce feline-associated tularemia, and cat owners should protect against bites/scratches and limit their pet’s outdoor access.

*Francisella tularensis*, a Tier 1 select agent, is one of the most pathogenic bacteria known and the etiologic agent of the zoonotic disease tularemia (*1*,*2*). Although the various subspecies of *F. tularensis* share considerable genomic content (>97% nt identity), they exhibit different degrees of virulence (*3*). *F. tularensis* subspecies *tularensis* (also known as type A) and subspecies *holarctica* (also known as type B) infections can be life-threatening if untreated (*1*). Type A is further subdivided into subtypes A.I and A.II; clade A.I contains strains that are associated with considerably higher death rates among humans than are the other members of this pathogenic species (*4*). Type B infections occur throughout the Northern Hemisphere, whereas type A infections occur primarily in North America (*3*).

Fatal and nonfatal cases of tularemia in domestic cats (*Felis catus*) have been reported, as has the transmission of this disease from cats to humans (*5*–*7*). Previous serologic surveys from several geographic regions determined that 12% of the domestic cats examined had antibodies to *F. tularensis* (*8*). Cases of feline-associated tularemia in humans continue to appear in the literature. However, the relative contribution of this source of *F. tularensis* transmission to humans is unknown and may be underrecognized. This study was conducted to examine the relative proportion and characteristics of feline-associated *F. tularensis* isolates within the repository at the University of Nebraska Medical Center (UNMC).

## The Study

A review was conducted of the 106 wild-type *F. tularensis* isolates voluntarily deposited in the UNMC collection during 1998–2012. These isolates were obtained from infected humans, animals, or ticks predominantly residing in Nebraska; however, isolates from several other regions were also included. Wild-type *F. tularensis* isolates had been transferred to UNMC from other locations, according to requirements of the national Select Agent Program (*2*). None of these isolates had been solicited from veterinary or environmental reference laboratories. Species identity for locally detected wild-type *F. tularensis* isolates to which humans had been exposed was confirmed by the Nebraska Public Health Laboratory in Omaha. Viable culture material was manipulated by authorized persons following select agent–approved Biosafety Level 3 criteria. For genotyping, we used the PCR-based differential insertion sequence amplification (DISA) method with the CR10 C+L+S primer set and pulsed-field gel electrophoresis (PFGE) of *Pme*I-digested *Francisella* spp. DNA, as previously described (*9*).

Of the 106 wild-type *F. tularensis* isolates in the repository, 54 (51%) were from humans for whom the source of exposure was unknown or undocumented, 29 (27%) were from cats (21) or from humans (8) with tularemia linked to infected cats, 5 (5%) were from humans with tularemia linked to infected ticks, 1 (1%) was from a human with tularemia linked to an infected rabbit, 16 (15%) were from animals with tularemia, and 1 (1%) was from an unknown host. Of the 29 isolates derived from feline-associated cases, 28 were associated with domestic cats and 1 involved a feral cat (Figure). Eight cases of human tularemia occurred through a cat bite: 7 of these cases involved adults and 1 involved a 6-year-old child. In 2 cases, the person was bitten while taking a rabbit from a cat. None of the 8 humans died.

**Figure:**
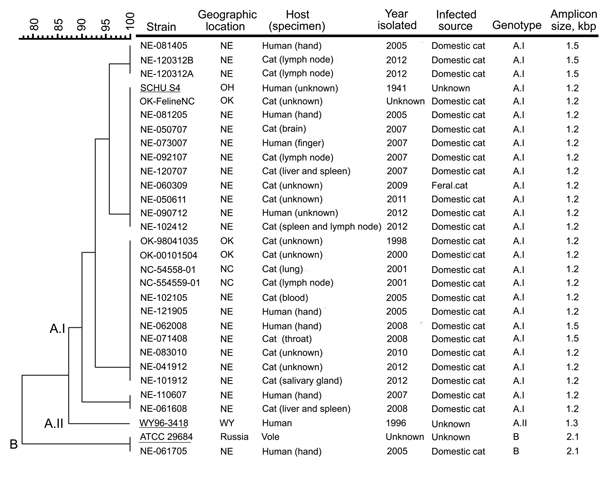
Molecular genotyping of *Francisella tularensis* isolates obtained from infected cats or humans bitten by an infected cat, United States, 1998–2012. Genotyping was performed by using pulsed-field gel electrophoresis (PFGE) and the PCR-based differential insertion sequence amplification (DISA) assay. A dendrogram of PFGE patterns obtained with *Pme*I-digested *F. tularensis* isolates is shown on the left; the scale bar at the top indicates distance in relative units. The genotype-specific amplicon lengths obtained with the DISA CR10 C+L+S primer set are shown on the right. Migration profiles of the *Pme*I restriction fragments from *F. tularensis* chromosomal DNA were normalized to *Sma*I-digested *Staphylococcus aureus* NCTC 8325 by using BioNumerics software (Applied Maths, Inc., Austin, TX, USA). Cluster analysis was performed by using the Dice correlation coefficient and the unweighted pair group mathematical average (UPGMA) clustering algorithm in the BioNumerics software. The DISA CR10 C+S primer pair identified the subtype A.I strains, whereas the CR10 C+L primer pair differentiated the subtype A.II and type B strains by the size of the amplicon produced. Underlining indicates *F. tularensis* strains SCHU S4 (subtype A.I), WY96–3418 (subtype A.II), and ATCC 29684 (type B), which were included as references. *F. tularensis* isolates NE-062508 and NE-073009 were no longer viable after storage and therefore not genotyped; these pathogens were isolated from domestic cats from eastern Nebraska (NE) in 2008 and 2009, respectively. ATCC, American Type Culture Collection; NC, North Carolina; OH, Ohio; OK, Oklahoma; WY, Wyoming.

We also analyzed a subset of the 29 *F. tularensis* isolates obtained from cats or humans bitten by cats. All but 2 of the 29 isolates were available for genotyping. DISA and PFGE results showed that 1 type B and 26 subtype A.I strains were responsible for the 27 cases of tularemia (Figure). Of the 8 cat bite–associated human tularemia cases, 1 was caused by a type B and 7 by subtype A.I strains. None of the feline-associated tularemia cases were caused by a subtype A.II strain.

PFGE demonstrated that the feline-derived *F. tularensis* A.I strains could be further divided into 4 subpopulations. For strains in 1 of these subpopulations, DISA results showed chromosomal polymorphisms in which a 1.2-kbp or a 1.5-kbp amplicon was produced. This finding is consistent with 2 unique origins for the strains, even though they were obtained from the same geographic area.

Subset analysis of Nebraska cases showed that 48% (24/50) of the wild-type *F. tularensis* isolates were feline-associated, and most of those 24 isolates (71%, 17/24) were associated with tularemia cases that occurred in 1 city in eastern Nebraska. The highest number of tularemia cases in Nebraska was reported in 2012, a year noted for extreme heat and drought conditions with warm weather beginning in early January and lasting through December. No feline-associated tularemia cases in Nebraska were reported during 1998–2004; however, 18 were reported during 2005–2011.

## Conclusions

Two different molecular methods demonstrated that *F. tularensis* subtype A.I was responsible for most of the tularemia cases in cats (96%, 26/27); 1 of the 27 cases was caused by a type B strain. This finding is consistent with subtype A.I strains being found predominantly east of the 100th meridian in the United States, whereas A.II strains appear to be restricted to the west of this meridian (*4*). More importantly, this study confirms that the highly virulent A.I strains are frequent causes of tularemia in cats and humans, a finding that is in agreement with those in a previous report (*4*), and that the type B clade can cause disease in these hosts.

Although cats are a well-known vector of *F. tularensis*, the strikingly high percentage of disease associated with cats in Nebraska was unexpected (48% in Nebraska vs. 27% in the UNMC repository). Of note, the highest number of feline-derived tularemia cases occurred in a city known to have multiple feral cat colonies and during a year with extended warm weather and extreme drought conditions. Warmer temperatures can accelerate the tick life cycle, augmenting the potential for transmission of vector-borne diseases (*10*,*11*), and droughts can contribute to impaired host immunity, thereby increasing the likelihood for the transmission of zoonotic pathogens at the limited sources of water (*12*). Therefore, although the natural reservoir of *F. tularensis* is unknown, we speculate that these environmental conditions exacerbated the spread of tularemia by increasing the abundance of ticks harboring and transmitting *F. tularensis* to outdoor animals. Nevertheless, the high relative contribution of cats to disease may be due to underrecognition of an existing problem, a new emerging threat caused by environmental factors, and/or reporting or other biases.

A 2012 systematic review reported that free-ranging domestic cats are probably responsible for the greatest percentage of bird and mammal deaths (*13*). This information and the findings from the current study support the supposition that although cats are considered incidental hosts for *F. tularensis*, their inherent predatory behavior increases the likelihood that they will acquire tularemia through the consumption of infected animals and exposure to contaminated ticks, animals, or water harboring this pathogen. A vaccine for tularemia is not available in the United States, and the overall prognosis for infected persons is poor if antibiotic drug treatment is not administered early in the infection process. Therefore, cat owners should protect themselves against potential bites and/or scratches and consider limiting outdoor access for domestic cats. In addition, because tularemia is often initially misdiagnosed (*14*), confirmatory testing by public health laboratories is important and will provide critical surveillance information regarding *F. tularensis* in the environment.

The high percentage of feline-associated tularemia cases reported in this study demonstrates that outdoor domestic cats are frequent vectors of *F. tularensis*, particularly in areas where the bacterium is endemic. The cases reported in this study were primarily from urban areas with nearby access to veterinarians and with cat owners/caretakers motivated to treat infected animals; therefore, our findings may not accurately estimate the prevalence of this pathogen within the feral and rural domestic cat populations. These findings do emphasize the importance of taking appropriate measures to reduce the transmission of feline-associated tularemia to humans and the need for a vaccine that protects against the highly virulent pathogen *F. tularensis*.
